# Supplementary material for: Drosha drives the formation of DNA:RNA hybrids around DNA break sites to facilitate DNA repair
Source: Nat Commun. 2018 Feb 7;9:532. doi: 10.1038/s41467-018-02893-x (PMC5803274; doi:10.1038/s41467-018-02893-x)
Supplement: Supplementary file 1 — Supplementary Information [file 41467_2018_2893_MOESM1_ESM.pdf]

Supplementary Materials for

Drosha drives the formation of DNA:RNA hybrids around DNA break sites to facilitate DNA repair

Wei-Ting Lu‡[1], Ben R Hawley‡[1], George L Skalka[1], Robert A Baldock†[2], Ewan M Smith[1], Aldo S Bader[1], Michal Malewicz[1], Felicity Z Watts[2], Ania Wilczynska[1] and Martin Bushell*[1]

*Correspondence to: mb446@leicester.ac.uk; ‡ Wei-Ting Lu and Ben R Hawley contributed equally.

[1]MRC Toxicology Unit, Lancaster Road, Leicester LE1 9HN, UK. [2]Genome Damage and Stability Centre, School of Life Sciences, University of Sussex, Brighton, UK. †Current address; University of Pittsburgh Cancer Institute, University of Pittsburgh, Pittsburgh, Pennsylvania, USA.

Supplementary Figure 1

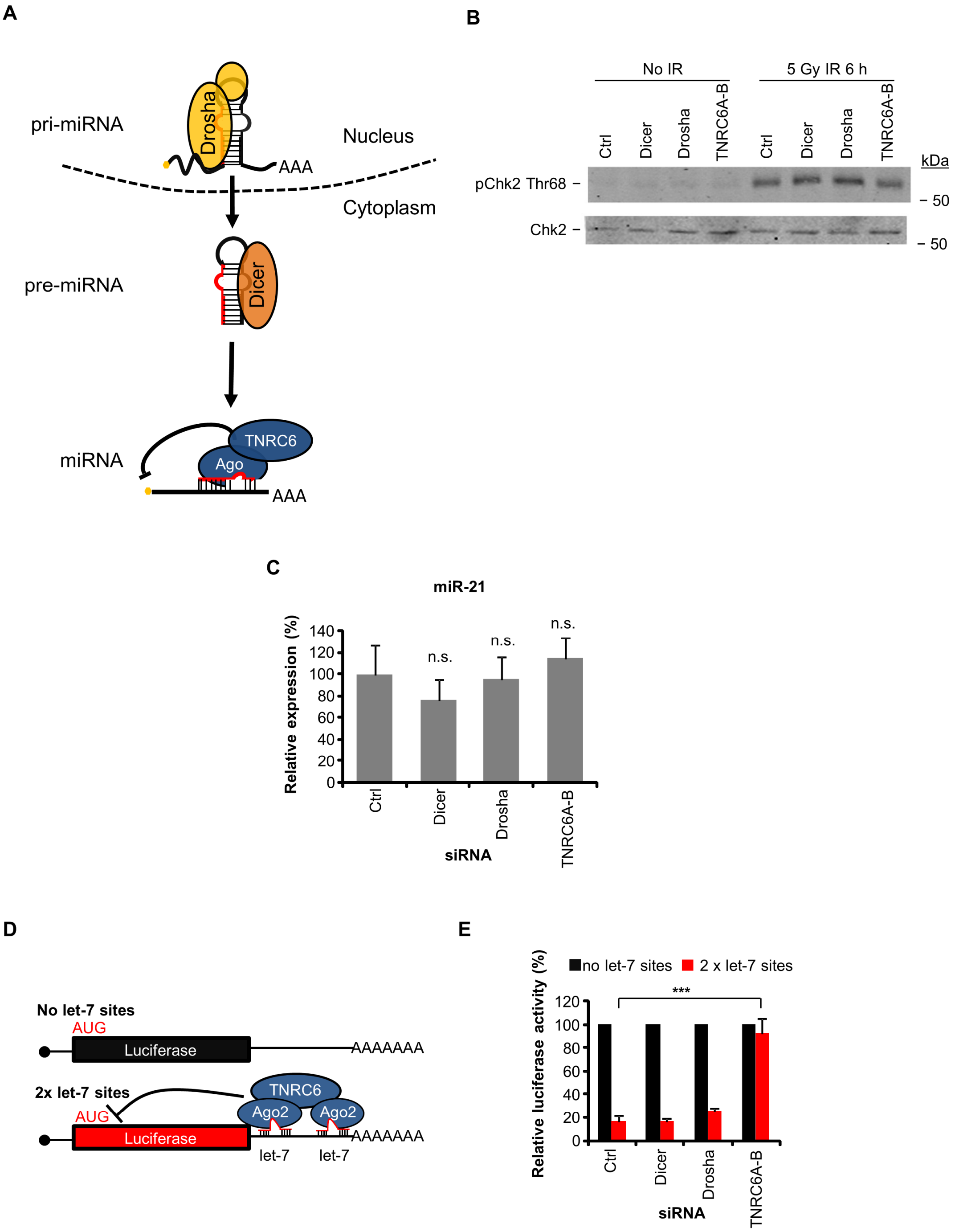

Supplementary Figure 1.

48 hour knockdown of Dicer and Drosha does not affect the canonical miRNA pathway in A549 cells.

(A) Cartoon outlining the miRNA biogenesis pathway in humans. The pri-miRNA hairpin-containing transcript is recognised and processed into pre-miRNA by Drosha. The stem loop is then exported to the cytoplasm, cleaved by Dicer into the mature double stranded miRNA, and loaded into Argonaute protein complexes. TNRC6 proteins are required for miRNA-mediated repression of target mRNAs[12].

(B) In A549 cells, the knockdown of Dicer, Drosha, or TNRC6A-B does not affect Chk2 phosphorylation at Thr68. This panel shows additional representative western blots from the same experiment as Fig.1C investigating the activation status of checkpoint protein Chk2, after DNA damage.

(C) The level of miR-21 miRNA relative to U6 snRNA in A549 cells does not significantly change upon the knockdown of Drosha, Dicer, or TNRC6A-B. N=3 biological replicates, 2-tailed 2-sample T-test.

(D) Cartoon depicting miRNA luciferase constructs used in (E).

(E) A549 cells were transfected with siRNAs as labelled, then co-transfected 24 hours later with Renilla luciferase reporter constructs and the firefly luciferase control pGL3 plasmid. Luciferase activity was assayed 24 hours later and the relative activity of the Renilla luciferase reporter (with or without let-7 miRNA target sites) to control firefly luciferase measured. The graph shows the expression of the let-7 reporter normalised to the no let-7 site control. The knockdown of TNRC6A-B, but not Dicer or Drosha, significantly abrogates the activity of

the miRNA pathway resulting in the de-repression of the let-7 reporter. Histogram is a representation of 3 biological replicates, *** $p \leq 0.001$, 2-tailed 2-sample T-test.

Supplementary Figure 2

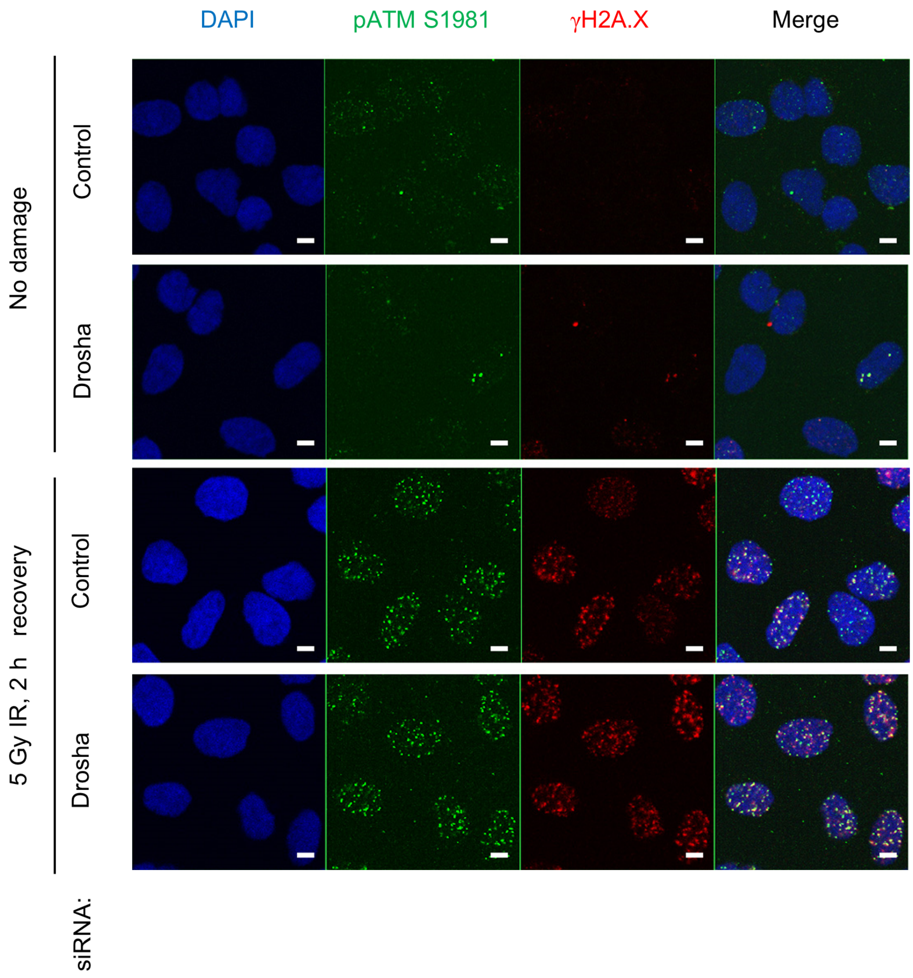

Supplementary Figure 2.

Drosha knockdown does not impair phospho-ATM or γH2A.X foci formation within 2 hours of IR. Representative immunofluorescence supporting Fig. 2A and 2B. Immunofluorescence of IR-induced pATM Ser1981 in A549 cells, 2 hours after 5 Gy IR. pATM foci are in the green channel, γH2A.X in red, DAPI-stained nuclei in blue. Cells without IR damage shown as a negative control. Scale bar, 10 µm.

Supplementary Figure 3

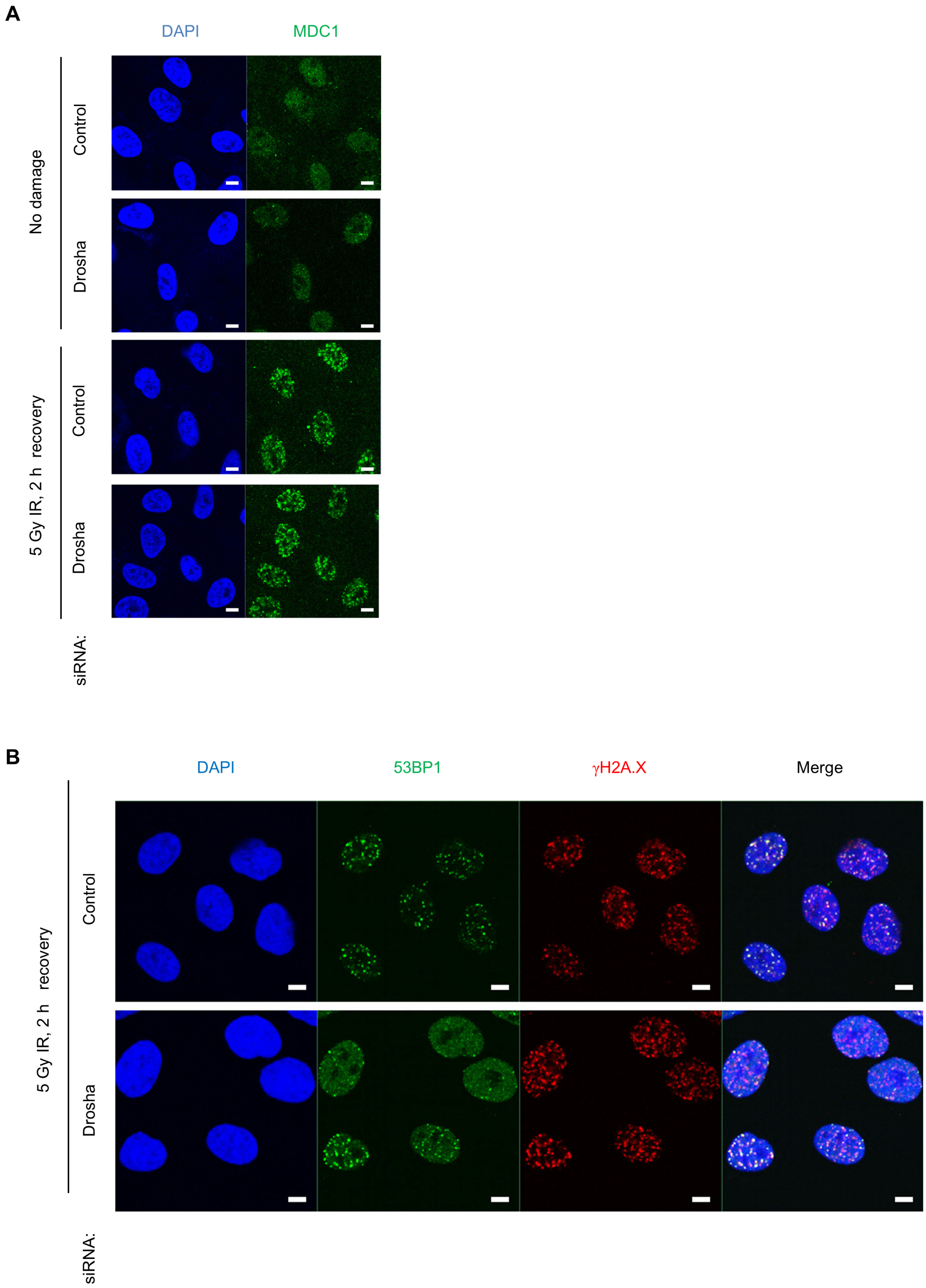

Supplementary Figure 3.

Drosha knockdown impairs 53BP1 foci formation but not MDC1 foci formation after IR. Representative images for Fig.2A and 2B. Representative immunofluorescence image of IR-induced **(A)** MDC1 and **(B)** 53BP1 foci in A549 cells, 2 hours after 5 Gy IR. Scale bar, 10 μm.

Supplementary Figure 4

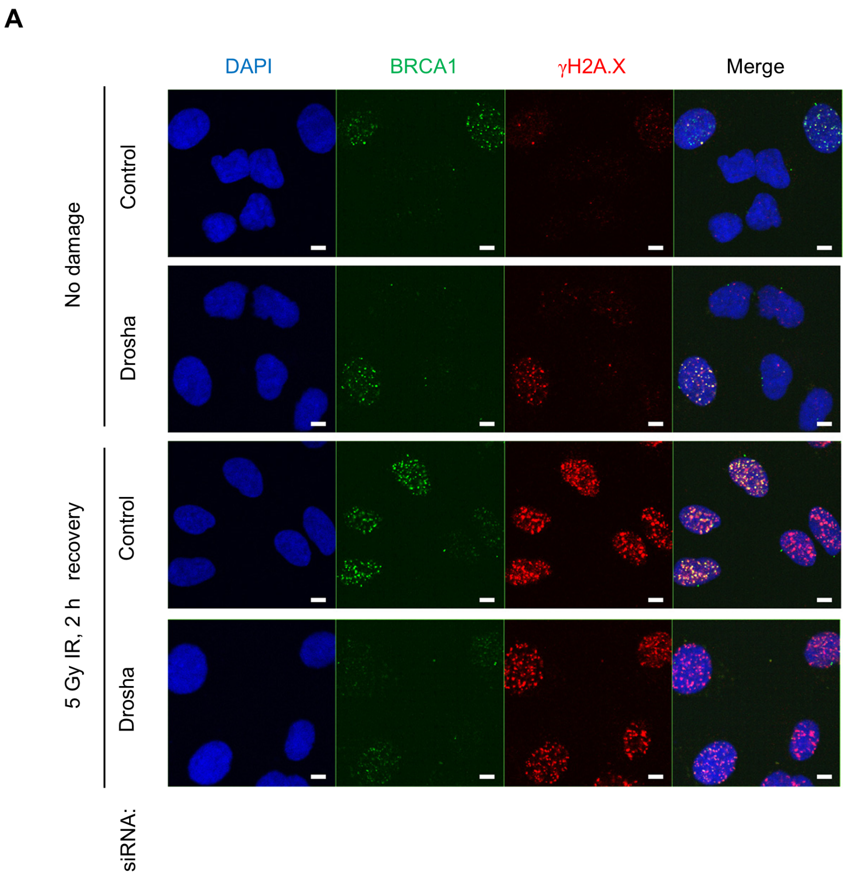

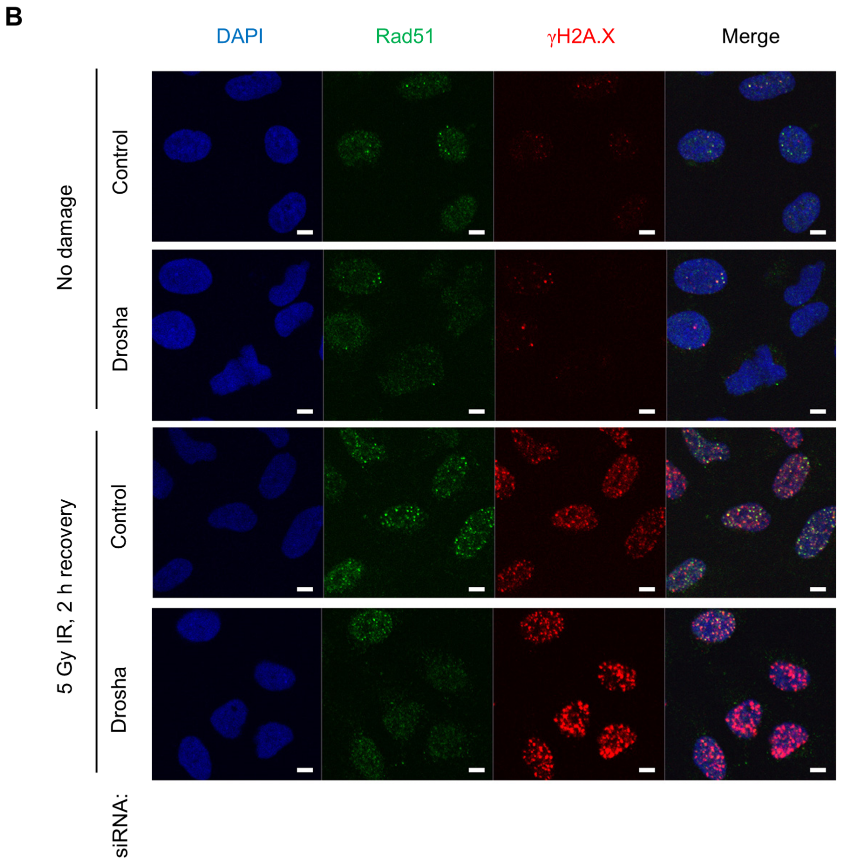

Supplementary Figure 4.

Drosha knockdown significantly impairs BRCA1 and Rad51 foci formation after IR. Representative immunofluorescence image visualizing IR-induced **(A)** BRCA1 foci or **(B)** Rad51 foci in A549 cells, 2 hours after 5 Gy IR. BRCA1/Rad51 foci are in green channel and γH2A.X in red. Scale bar, 10 μm.

Supplementary Figure 5

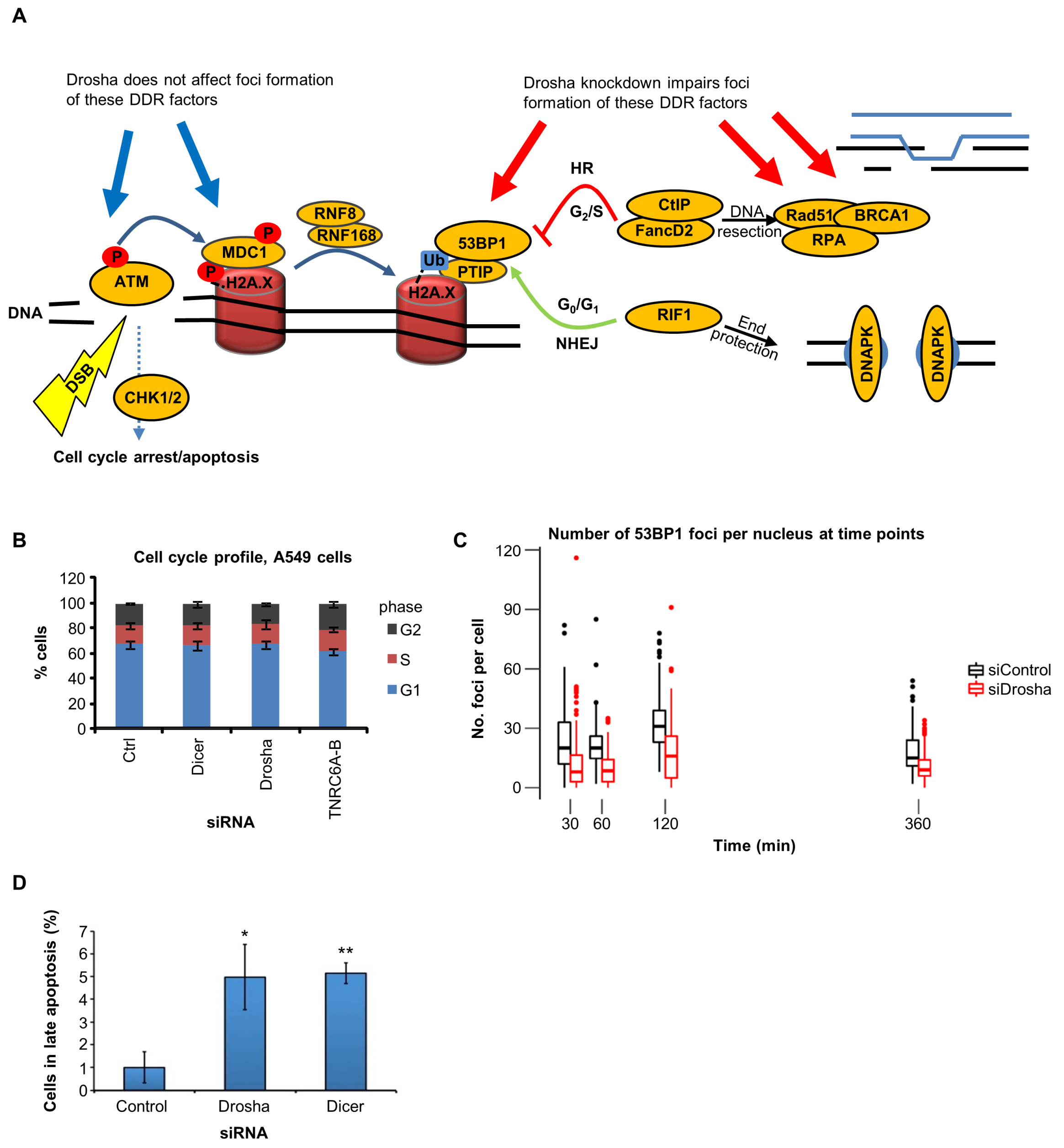

Supplementary Figure 5.

Depletion of Drosha results in a reduction of 53BP1 foci at multiple time points and increased apoptotic entry.

(A) Cartoon showing steps in DNA damage response and whether they are affected by Drosha knockdown as determined in this study. Drosha knockdown does not affect localization of pATM, MDC1 and γH2A.X at DNA damage sites (blue arrows). In contrast, red arrows point to steps in DDR impaired by Drosha depletion, including 53BP1, Rad51 and BRCA1 foci formation.

(B) Cell cycle status of A549 cells is not significantly affected by Dicer, Drosha or TNRC6A-B knockdown. A549 cells were transfected with siRNA for 48 hours. Cells were then collected, fixed and stained with propidium iodide. Flow cytometry was used to analyse cell cycle status. N=3 biological repeats, 2-tailed 2-sample Student's T-test.

(C) The number of 53BP1 foci per nucleus was counted in control or Drosha siRNA treated cells fixed at each of the indicated time points following 5 Gy IR. Quantitation was performed on relevant experiments in Fig 1, 2 and 3 (plus 60 mins timepoint) using the ImageJ FindFoci plugin, as described in Fig. 1.

(D) Proportion of cells entering late apoptosis. U2OS cells were initially treated with the indicated siRNA for 48 hours before addition of 1 µg/ml of the radiomimetic damage agent bleomycin for 24 hours. Media, wash, and adherent cells were all harvested and stained with Annexin V-FITC/DRAQ7. Apoptotic cell populations were determined by flow cytometry analysis. Shown is the percentage of cells in late apoptosis following normalization to the control siRNA condition. N=4, Student's paired t-test, *p≤0.05, **p≤0.01.

Supplementary Figure 6

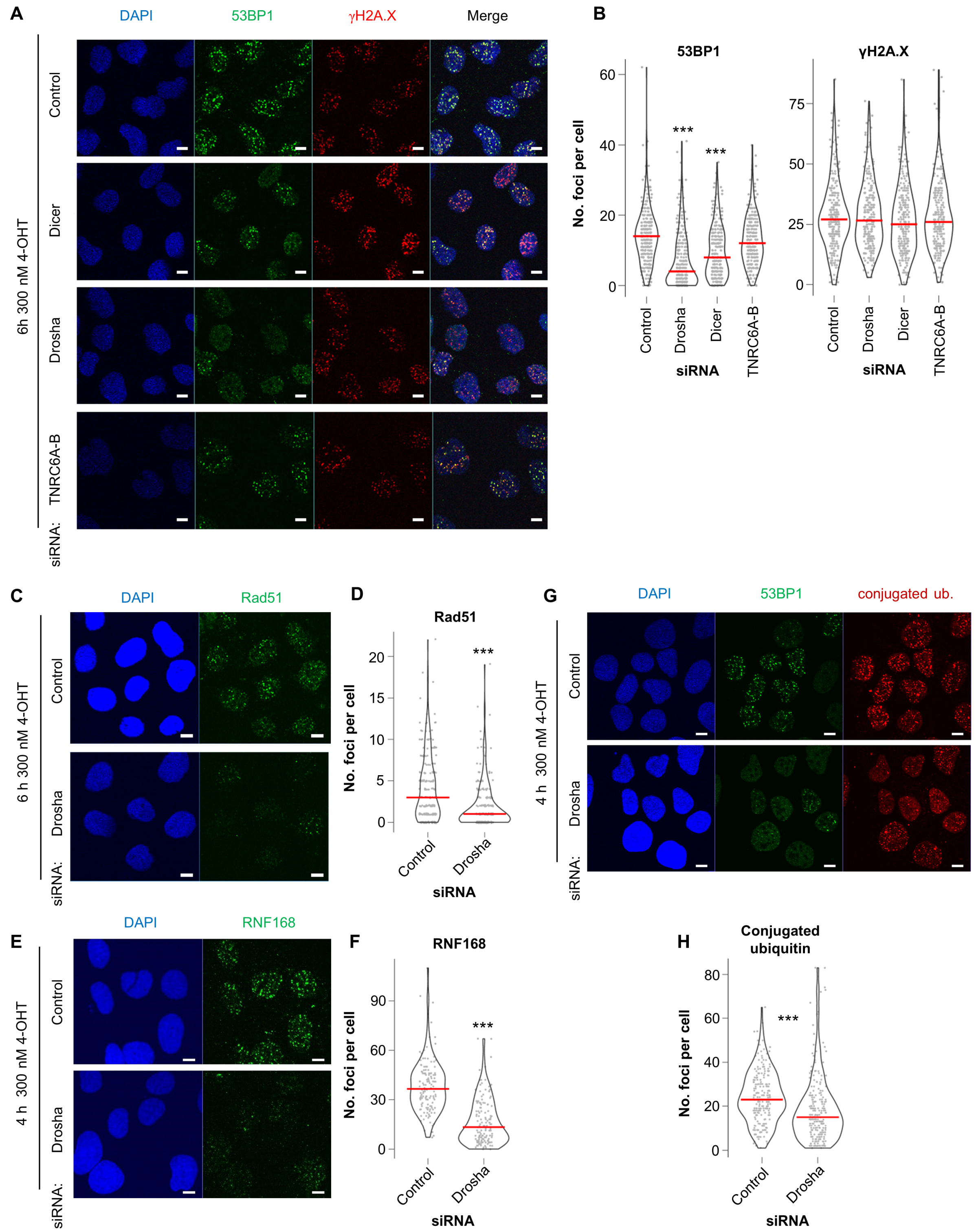

Supplementary Figure 6.

Knockdown of Dicer or Drosha, but not TNRC6A-B, impairs DNA damage induced foci formation in the U2OS-HA-ER-AsiSI system. (A) Representative immunofluorescence image visualizing damage-induced 53BP1 foci in U2OS-HA-ER-AsiSI cells, 6 hours after 300 nM 4-OHT addition, 53BP1 in green channel and γH2A.X in red. Scale bar, 10 µm. **(B)** Quantification of DDR foci shown in (A). The number of 53BP1 or γH2A.X foci per nucleus was counted using the FindFoci ImageJ plugin and plotted as individual data points and a violin plot across 3 biological replicates each counting >60 cells per condition. Red line represents the median in each condition. Statistical testing performed using Dunn's test with Bonferroni correction for multiple comparisons, ***$p \leq 0.001$. **(C)** Immunofluorescence image of damage-induced Rad51 foci in U2OS-HA-ER-AsiSI cells, 6 hours after 300 nM 4-OHT addition. Scale bar, 10 µm. **(D)** Quantification of DDR foci shown in (C), done as in (B). Statistical testing was performed using the Mann-Whitney non-parametric test, ***$p \leq 0.001$. **(E)** Immunofluorescence of RNF168 foci 4 hours following 300 nM 4-OHT addition in U2OS-HA-ER-AsiSI cells. Scale bar, 10 µm. **(F)** Quantification of (E) performed as in (B) and (D). Statistical testing was performed

using the Mann-Whitney non-parametric test, ***p≤0.001. **(G)** Representative immunofluorescence image showing 53BP1 foci (green) and conjugated ubiquitin (FK2) (red) foci 4 hours following 300 nM 4-OHT addition. **(H)** Quantification of the number of conjugated ubiquitin (FK2) foci per cell, performed as in part (B). Statistical testing performed using the Mann-Whitney non-parametric test, ***p≤0.001.

Supplementary Figure 7

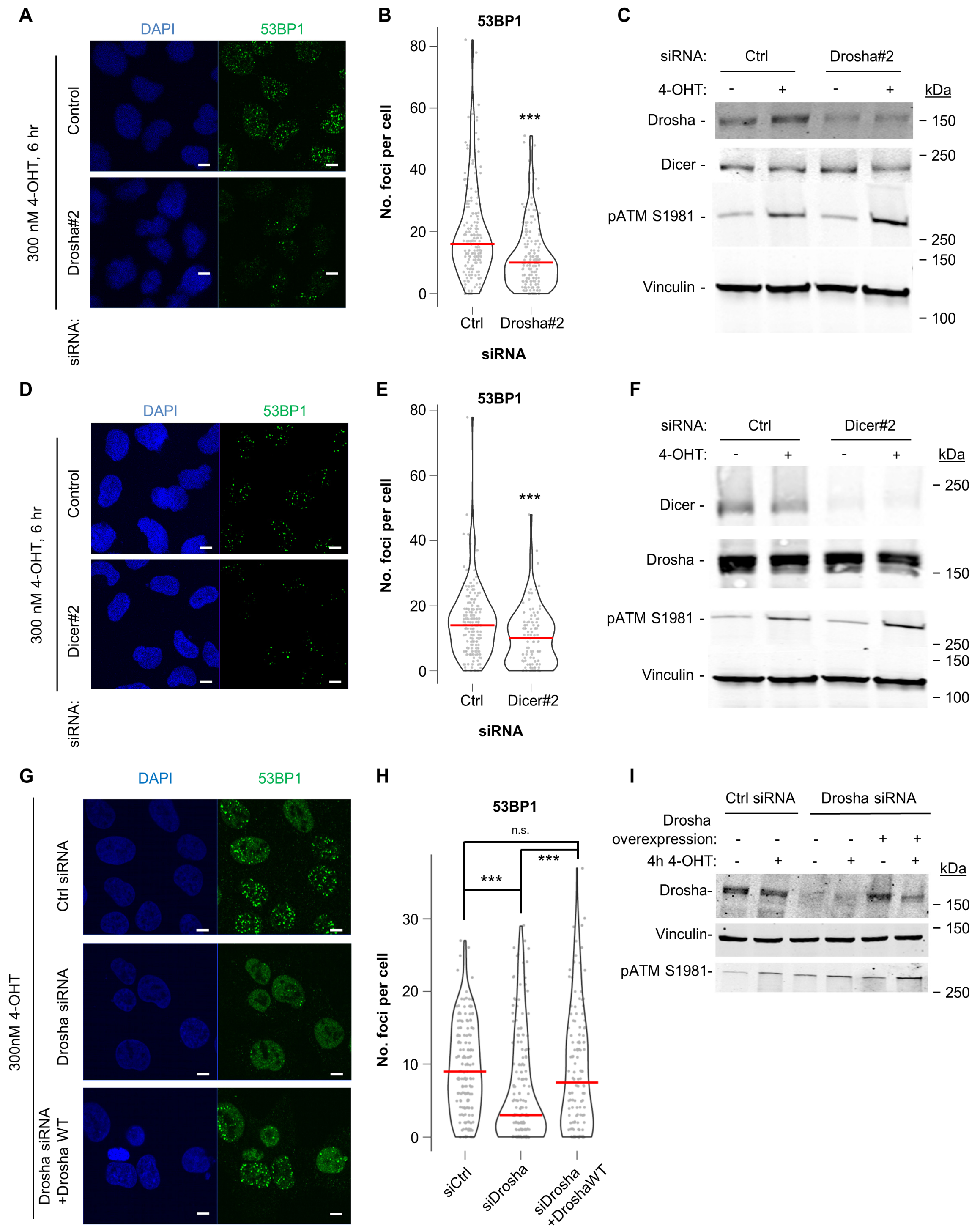

Supplementary Figure 7.

Use of alternative siRNA and rescue experiments confirm that the effects of Drosha and Dicer depletion are specific.

(A) Representative IF images of AsiSI-induced 53BP1 foci (green) using an alternative Drosha siRNA 6 hours after 4-OHT addition. Scale bar, 10 µm.

(B) Quantification of images in A. The number of foci per nucleus was counted using the Findfoci ImageJ plugin and plotted as individual data points and a violin plot across 3 biological replicates each counting >60 cells per condition. Red line represents the median in each condition. Statistical testing performed by Mann-Whitney non-parametric test, ***$p \leq 0.001$.

(C) Validation of siRNA knockdown.

(D, E, F) As for parts A-C using an alternative Dicer siRNA.

(G) Representative IF of AsiSI-induced 53BP1 foci (green) and DAPI-strained nucleus (blue) in U2OS-HA-ER-AsiSI cells treated with control or Drosha siRNA, with or without rescue by over-expression of siRNA-resistant Drosha. Scale bar, 10 µm. **(H)** Quantification of images in (G) as performed in (B) and (E). N=3, >60 cells per

biological replicate per condition. Red line represents median number of foci per nucleus, ***$p \leq 0.001$ Dunn's test with Bonferroni corrections for multiple comparisons. **(I)** Western blot validation of (G).

Supplementary Figure 8

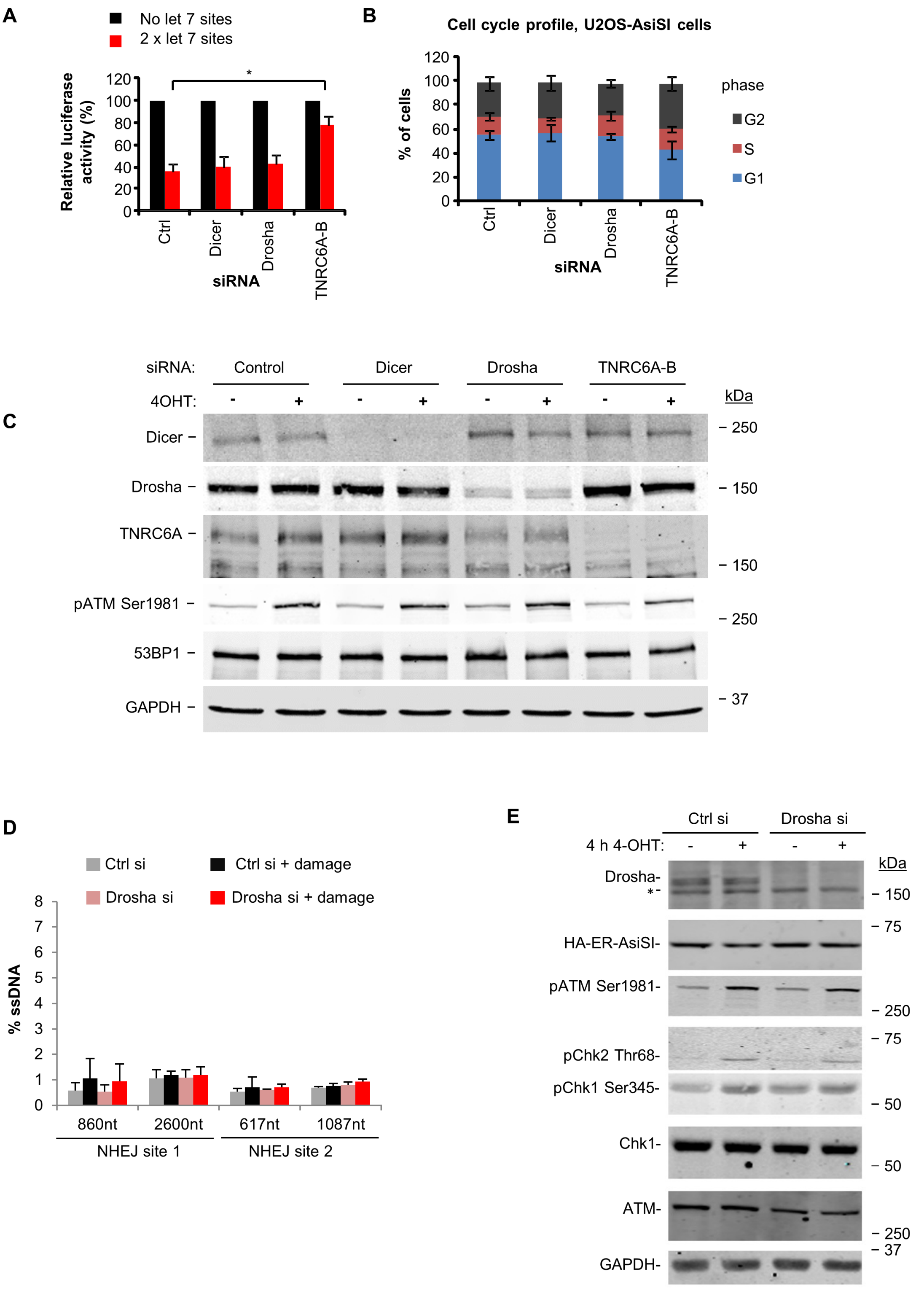

Supplementary Figure 8.

Knockdown of Dicer and Drosha does not affect miRNA activity and cell cycle status in the U2OS-HA-ER-AsiSI system.

(A) Only the depletion of TNRC6A-B, and not Dicer or Drosha proteins significantly impairs miRNA-mediated repression. Luciferase reporter assay as in Fig. S1D and S1E was carried out in U2OS-HA-ER-AsiSI cells N=3, error bars = SEM, * $p \leq 0.05$, 2-tailed 2-sample Student's T-test.

(B) Cell cycle status of U2OS-HA-ER-AsiSI cells is not significantly affected by Dicer, Drosha or TNRC6A-B knockdown. Experiment performed as in Fig. S5B. Graph represents 3 biological repeats, 2-tailed 2-sample Student's T-test.

(C) Representative western blot showing the siRNA knockdown efficiency of Dicer, Drosha, and TNRC6A-B proteins in U2OS-HA-ER-AsiSI cells. Activation of ATM protein by autophosphorylation confirms that the DNA damage response is activated after 6 hours of treatment with 300 nM 4-OHT.

(D) No significant resection of DNA is observed after DNA damage induction at two NHEJ sites with or without Drosha knockdown. Supplementary figure supporting Fig. 5B. NHEJ-repaired sites as reported in[7]. Results are of 3 biological repeats, error bars = SD, 2-tailed 2-sample Student's T-test.
(E) Drosha knockdown does not affect ATM or checkpoint signaling in U2OS-HA-ER-AsiSI cells. Representative western blot supporting Fig. 5B confirms Drosha knockdown and that the impairment of ssDNA is not due to defective checkpoint activation.

Supplementary Figure 9

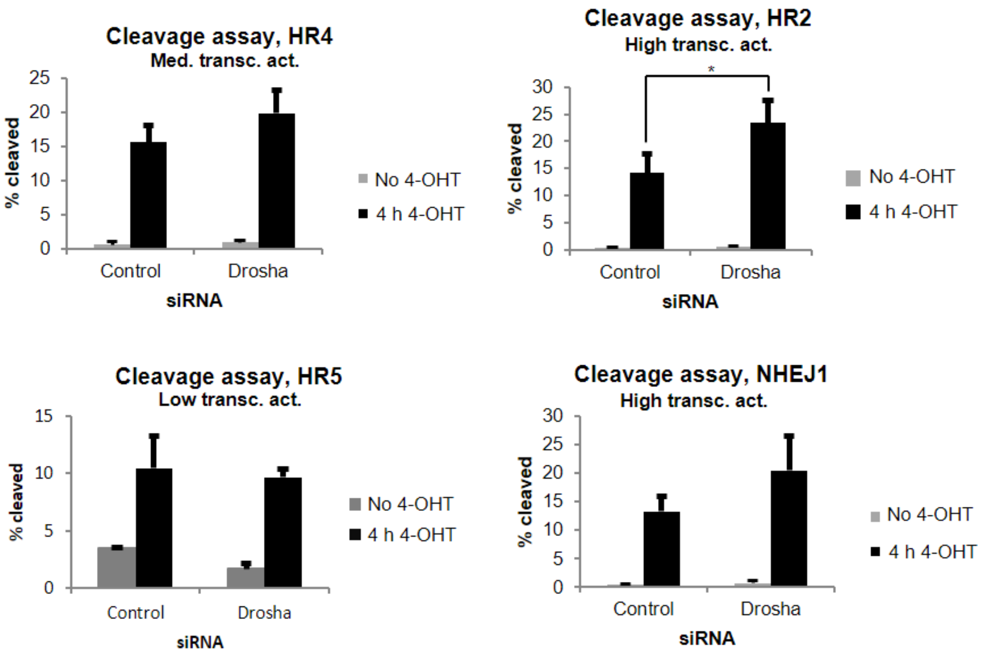

Supplementary Figure 9.

AsiSI cleavage assay.

The AsiSI cleavage assay was performed as in[7,73]. Briefly, genomic DNA was extracted from cells treated with or without 4-OHT for 4 hours. Biotinylated oligonucleotides with an AsiSI-compatible overhang were ligated to gDNA followed by EcoRI fragmentation of the gDNA. Ligated fragments were enriched using streptavidin beads and eluted by cleavage at the Hind-III site within the oligonucleotide linker. Cleavage percentage was calculated as the C_T value for pulldown divided by the C_T value for 10% input. This was normalized to the cleavage percentage for an *in vitro* cleaved plasmid that was spiked in prior to ligation to get an absolute measure of ligation-capable DNA ends. It should be noted that this method likely under-represents the true percentage of cutting due to modifications of the 5'-monophopshate ends *in vivo* and because already processed ends will not be detected. Error bars=SD, Student's 2-sample T-test, *$p \leq 0.05$, N=3. Each graph is annotated with its transcriptional activity, as assessed using our 4sU sequencing data (see Materials and Methods).

Supplementary Figure 10

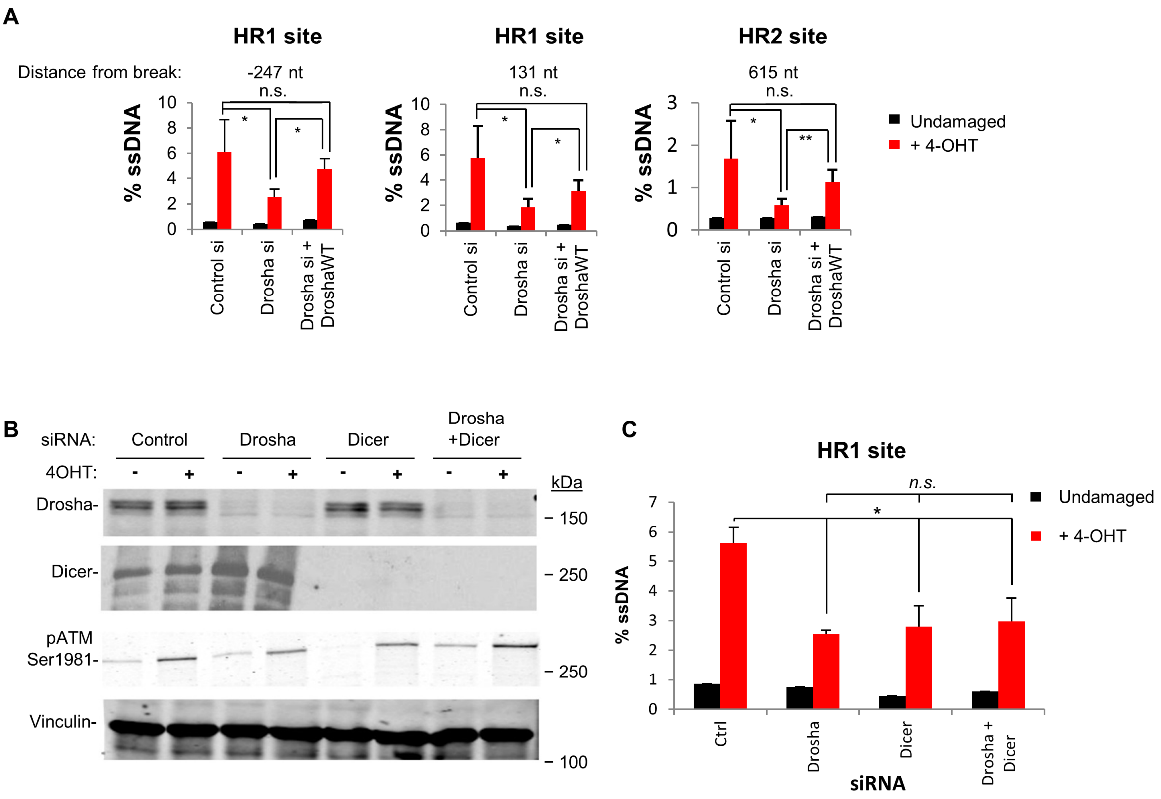

Supplementary Figure 10.

The double knockdown of Drosha and Dicer does not cause a stronger resection defect.

(A) Resection assay as in Fig. 5. Drosha depletion reduces resection at two HR prone loci (HR1 -247nt, 131nt; HR2 615 nt from break, respectively). This reduction is reversed when siRNA-resistant Drosha plasmid is nucleofected prior to siRNA transfection. Results are of at least 3 biological repeats, error bars =SD, 2-tailed 2-sample Student's t-test, *$p<0.05$, **$p<0.01$. Western blot showing the depletion and overexpression for this experiment is in Fig. S7I

(B) Western blot validation of knockdown of proteins by indicated siRNA. pATM is used as a positive control for damage induction.

(C) qPCR resection assay as in figure 5AB (HR1 site 131nt) following 4 hours of damage induction. Error bars=SD, Student's 2-sample T-test, *$p\leq0.05$, N=3.

Supplementary Figure 11

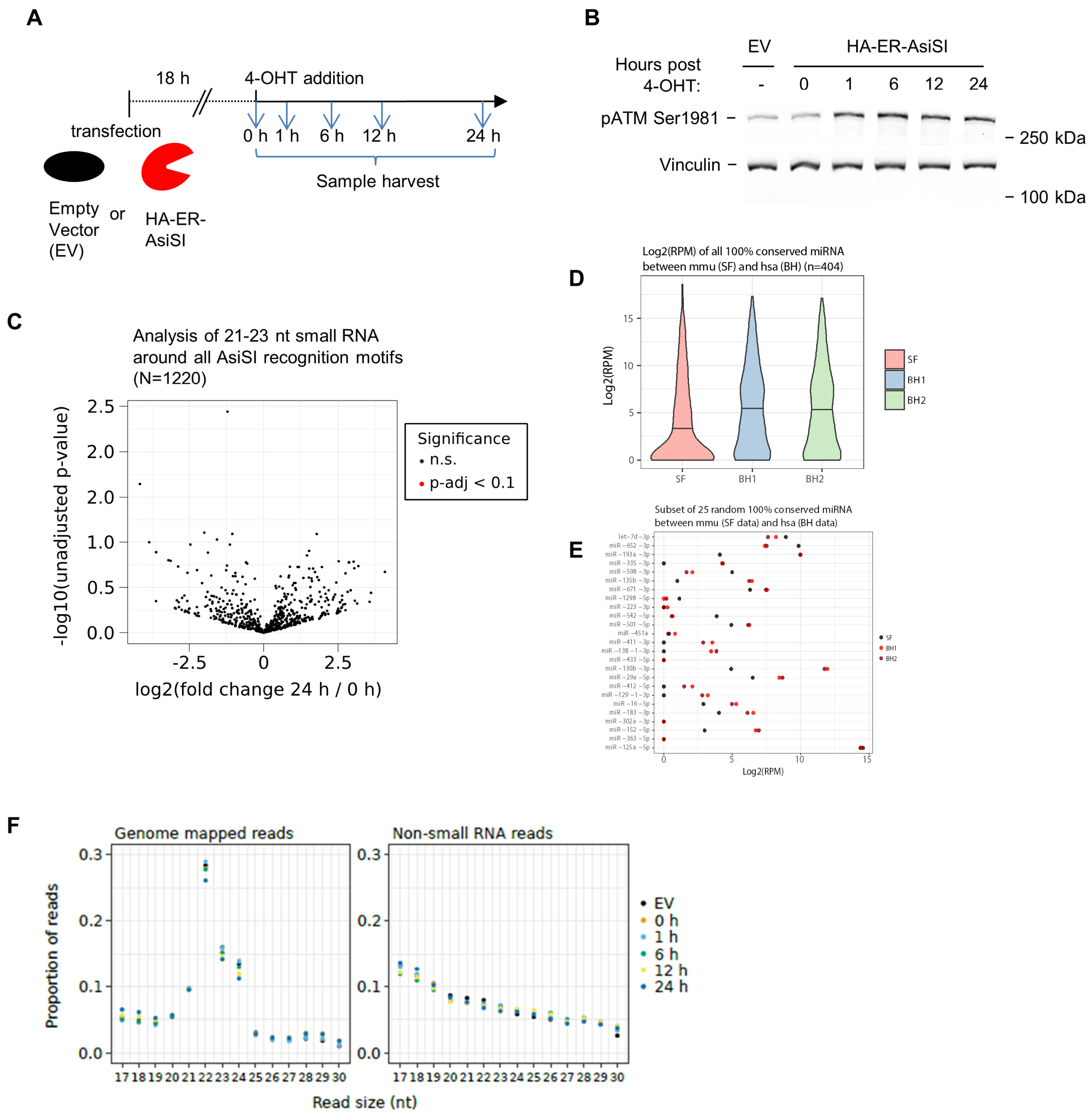

Supplementary Figure 11.

Next generation sequencing of small RNA in the U2OS cell line after induction of DNA damage.

(A) Schematic of the experimental set up. U2OS cells were transfected with either the HA-ER-AsiSI expressing plasmid or an empty vector (EV) by electroporation. 18 hours later, cells were incubated with 300 nM 4-OHT and harvested at the indicated time points. The 0 hour time point represents a sample where AsiSI was transfected but not treated with 4-OHT (i.e. undamaged). Small RNA extracted at the indicated time points was subjected to NGS.

(B) Autophosphorylation of ATM can be seen within 1 hour of treatment with 300 nM 4-OHT. Protein was harvested alongside RNA to validate damage induction.

(C) Statistical analysis reveals no significant increase of non-miRNA small RNA reads at any time point following damage induction around any potential AsiSI cut sites. Volcano plot of –log10(unadjusted p-value) against log2(fold change) comparing 21-23 nt non-miRNA small RNAs at 0 h (- 4-OHT) against the 24 h damage (+ 4-OHT) time point. Any differential expression with an adjusted p-value of less than 0.1 will be highlighted in red.

(D) Comparison of coverage of the miRNAs in the two small RNA-seq repeats used in this study (current study, CS.1 and CS.2) against the one in[14] (referred to as REF14) shows that the depth of our sequencing is at least as sensitive for low abundance small RNA as in previous studies. As REF14 data is from mouse cells, only 100% conserved miRNAs (by sequence) were compared (total 404 miRNAs). Raw reads were counted for each conserved miRNA and normalized to total reads per million (RPM). This was then plotted as a violin plot with median to show density of miRNA depth by RPM across each sample.

(E) A random subset of 25 miRNA from panel (D) to show RPM values for individual miRNAs. Points in red represent our datasets, while black points denote data from Francia *et al.*

(F) Size distribution of small RNA reads mapping to the genome before (left) and after (right) removal of all annotated small RNA (i.e. miRNA, Y RNA, snRNA, tRNA, etc) as described in the Methods section.

Supplementary Figure 12

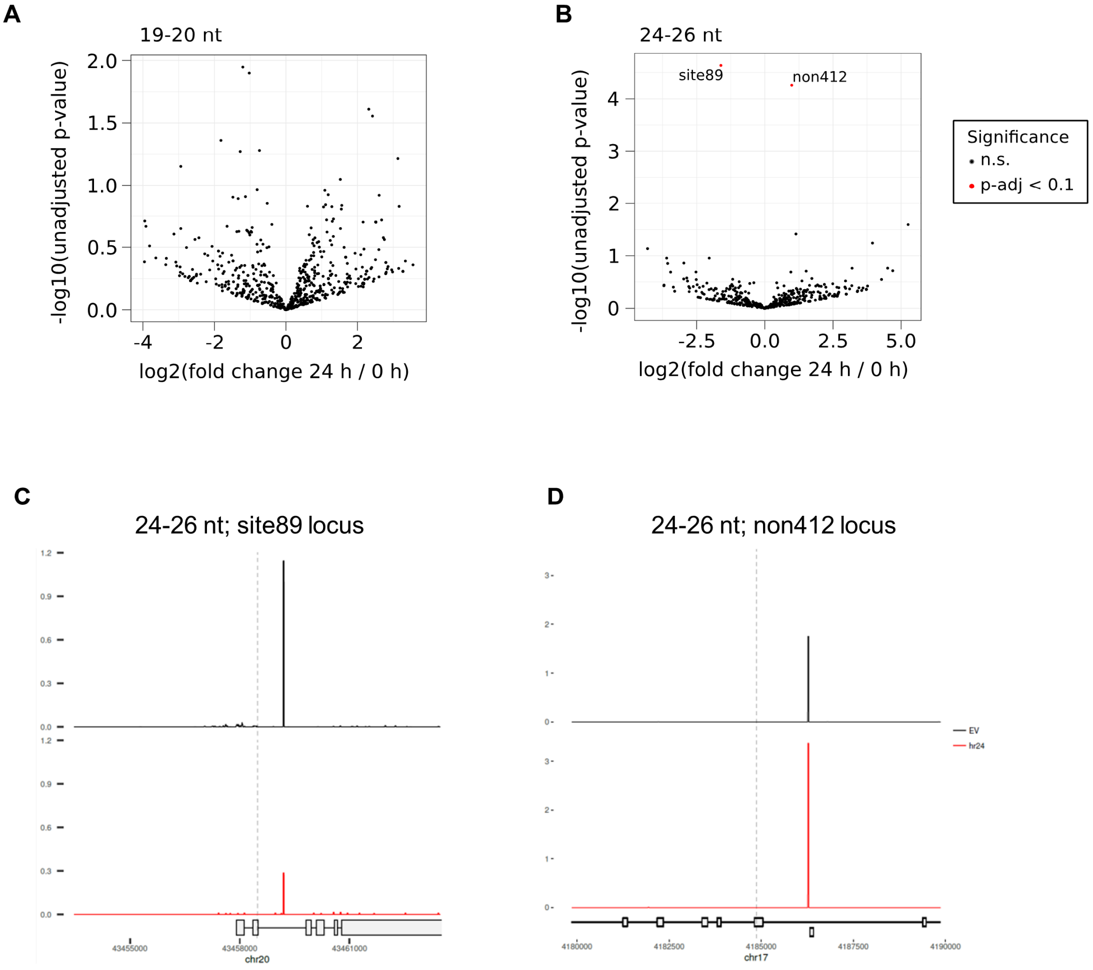

Supplementary Figure 12.

Analysis of small RNA of other size classes shows no evidence of damage-induced small RNA around AsiSI cut endogenous loci.

(A) Volcano plot showing outcome of analysis for small RNA of size 19-20 nt performed as in Fig. S11C. Reads not corresponding to annotated small RNA (e.g. miRNA) were counted at each AsiSI locus using HTSeq-count and analysed using DESeq2. Two GLM models were designed to analyse changes over time as well as at any individual time point. No loci passed significance threshold (FDR<0.1) at any time point. Shown here is the last time point: 24 hours post 4-OHT addition.

(B) As in (A) and figure S11C, analyzing 24-26 nt small RNA. Two loci (red, labelled) passed significance threshold (FDR<0.1), one known to be cut (site 89) and one uncut (site 412). Signal at the cut locus decreases upon damage whereas the uncut locus increases.

(C) 24-26 nt RNA read density around the site89 locus shows that reads pile up at one position within an intron of the AsiSI cut gene and is thus likely an unannotated small RNA species.

(D) 24-26 nt RNA read density at the non412 locus, which was not determined to be cut[7]. These reads pile up exclusively at a Y RNA, and this sequence was retained for the analysis likely as a result of misannotation.

Supplementary Figure 13

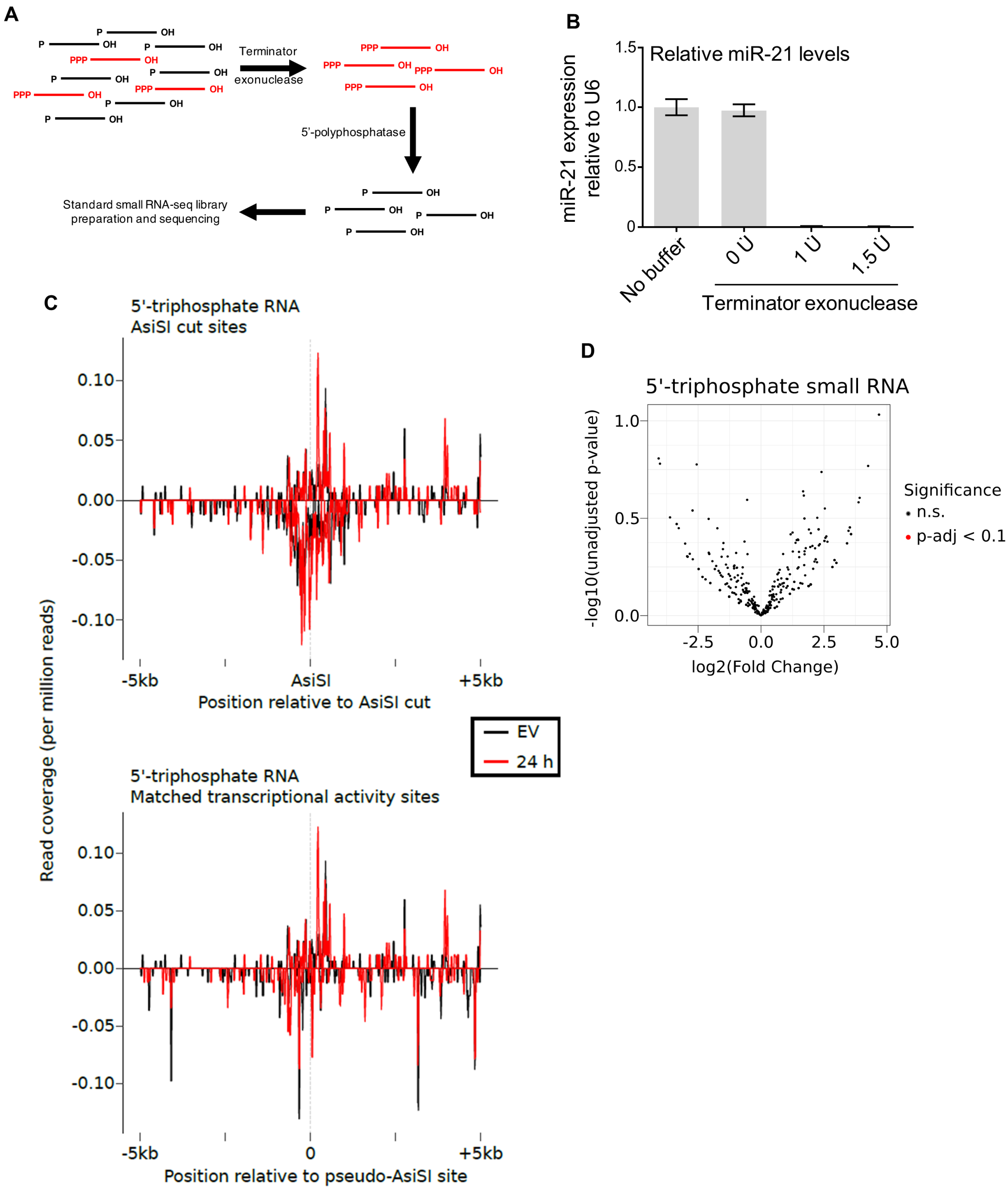

Supplementary Figure 13.

Sequencing of 5'-triphosphate small RNA species provides no evidence of damage-induced small RNA that could have been produced through an uncharacterized amplification pathway.

(A) Schematic of the approach used to first remove 5'-monophosphate RNA (e.g. miRNA) using Terminator exonuclease, followed by dephosphorylation of the remaining 5'-di/triphosphates with polyphosphatase. The resulting RNA is then used for standard library preparation. The empty vector (EV) and 24 hour 4-OHT samples from the standard small RNA-Seq experiment described in Fig. 6, S12, S13 was used.

(B) TaqMan qPCR analysis of the abundant miRNA miR-21 following Terminator exonuclease treatment confirms it successfully eliminates 5'-monophosphate RNA. miRNA abundance was normalized to snoRNA U6, as U6 is 5'-capped and thus unaffected by Terminator exonuclease.

(C) Read density of 5'-triphosphate small RNA analysed as in Fig. 6. Top panel, the aggregation of the 99 cut AsiSI loci; bottom panel, matched transcriptional activity control uncut loci.

(D) DESeq2 analysis of 5'-triphosphate RNA reads at the AsiSI recognition site loci as in figures S11C and S12AB.

Supplementary Figure 14

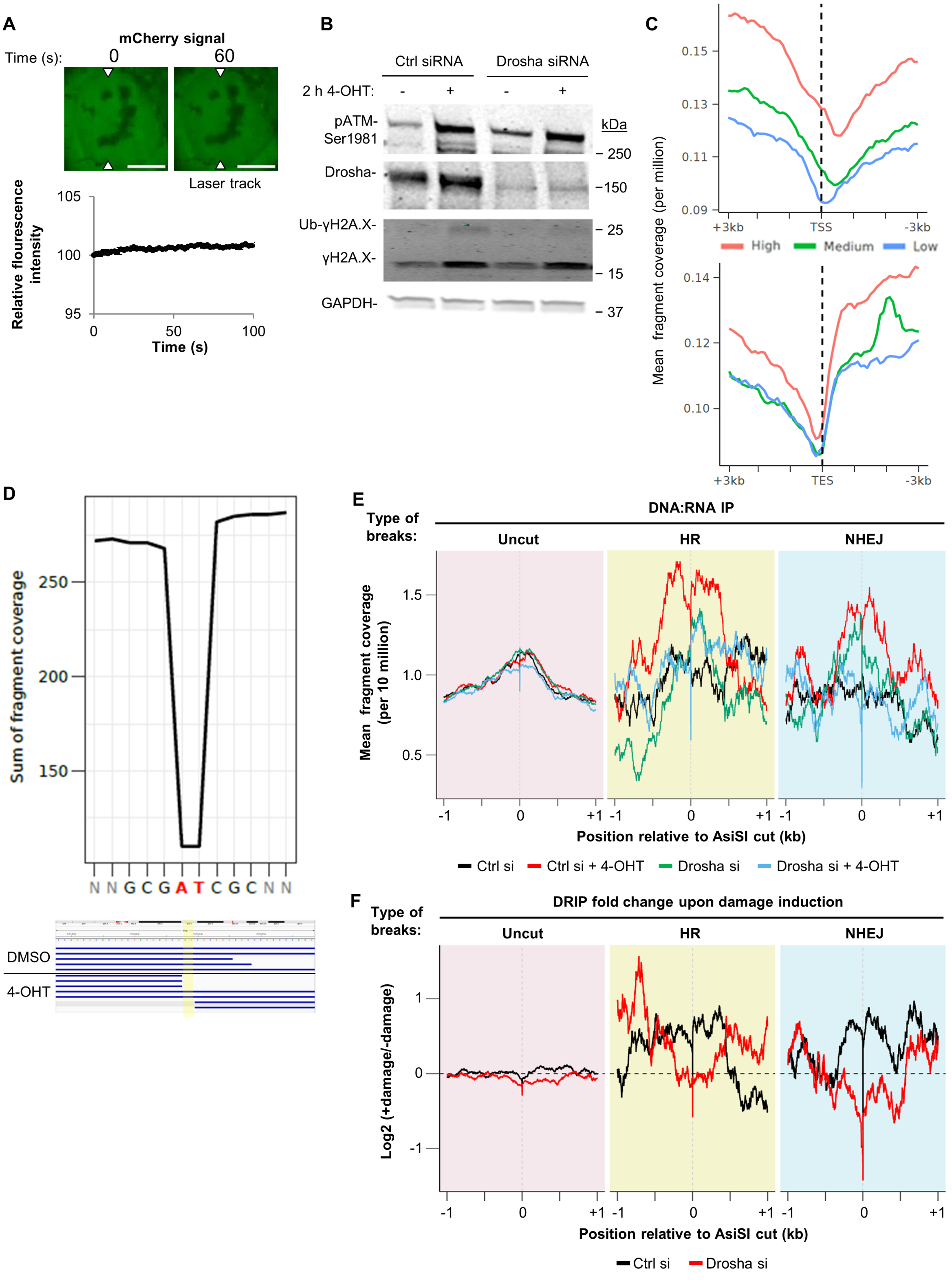

Supplementary Figure 14.

Drosha drives DNA:RNA hybrid formation around DNA break sites.

(A) Unlike inactivated mCherry-RNase H1 (Fig. 7A), negative control mCherry protein does not localize to DNA break sites after laser microirradiation. Arrows indicate position of laser track. Experiment was carried out in a similar manner to Fig. 7A. Representative fluorescence images, top. Scale bar, 10 µm. Bottom, histogram showing quantitation of mCherry signal after laser microirradiation, data is representive of 113 cells over 2 repeats. Error bars=SEM.

(B) Verification of Drosha depletion, and confirmation of DNA damage signaling activation (pATM and γH2A.X). U2OS-HA-ER-AsiSI cells with or without Drosha knockdown were induced for 2 hours with 300 nM 4-OHT.

(C) Metagene profiles showing DRIP fragment coverage around the transcription start site (TSS) and transcription end/termination site (TES) of all genes. All genes were split into equally-sized low, medium and

high groups based on TPM for each gene. Regions -/+3kb around each TSS and TES were binned into 200bp windows and DRIP fragment coverage was calculated in each.
(D) Base occupancy of DRIP fragments at the AsiSI recognition site shows that the end prep stage of library preparation cleaves off the AsiSI 3' overhangs. Per-base coverage of fragments was calculated and summed at the AsiSI site. Below is a screenshot of an IGV view of fragments around the *HUNK* AsiSI cut site, showing that DNA damage-induced DNA:RNA hybridization occurs in non-resected DNA proximal to the break site.
(E) Coverage plots around AsiSI cut sites, by repair type for all siRNA and damage conditions. Coverage per 10 million fragments of sequenced DNA:RNA immunoprecipitated fragments was calculated -/+ 1 kb either side of AsiSI sites by repair type or those sites that are not known to be cut[7]. HR sites n=25, NHEJ sites n=25, uncut n=1121. The mean per-base coverage was calculated within each set and plotted for control and Drosha siRNA, both undamaged and damaged.
(F) Comparison of the effect of Drosha depletion on the enrichment of DNA:RNA hybrids forming around AsiSI break sites. The log2 fold change of the per-base coverage after damage induction by 4-OHT is plotted for HR repaired sites (n=25), NHEJ repaired sites (n=25) and AsiSI recognition sites that are not cut in the cell (n=1121). A dashed line is drawn at y = 0 denoting no change after DNA damage; negative values denote sites that are depleted upon damage.

Supplementary Figure 15

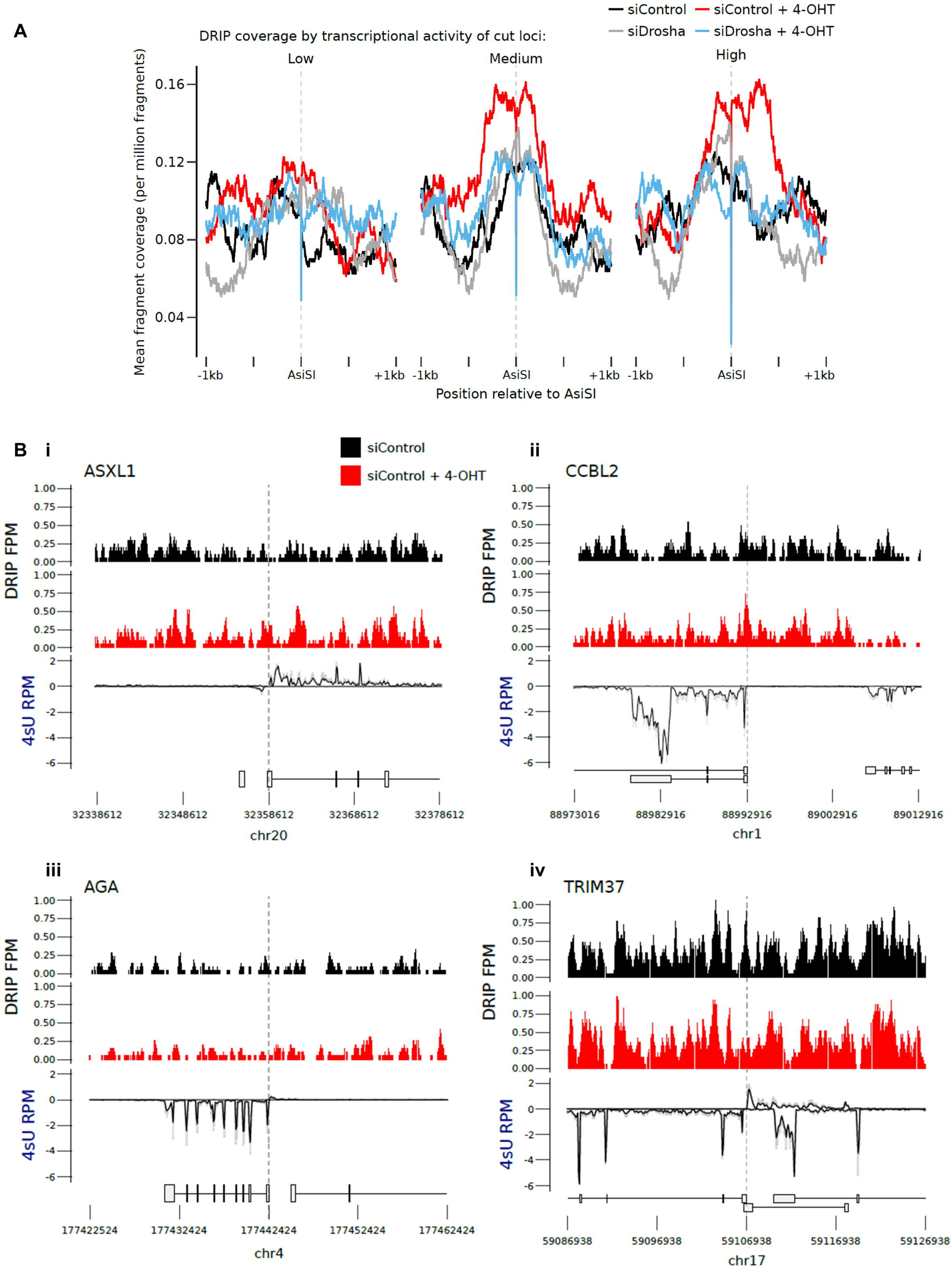

Supplementary Figure 15.

DRIP-seq fragment coverage around individual AsiSI cut sites shows that R-loop formation may be an active process.

(A) DRIP-Seq fragment coverage was calculated at each AsiSI locus, split into three equally sized subsets based on transcription activity. This was determined using the 4sU-Seq dataset described in the Materials and Methods section by determining transcripts per million (TPM) for each AsiSI-cut gene. As the 4sU-Seq dataset is specifically enriched for newly transcribed RNA in a 1 hour window, TPM for each gene was used as a readout of the transcriptional activity of that gene.

(B) Transcriptional activity is not the sole determinant of DDR-induced DNA:RNA hybridization. DRIP-Seq fragment (per million, FPM) coverage was calculated in 100 bp bins within a 40 kb region centred on individual AsiSI cut sites (dashed vertical lines), together with the 4sU-Seq reads (per million, RPM). **(i, ii)** AsiSI cuts within the *ASXL1* and *CCBL2* genes produce DNA:RNA hybrids at the cut site following induction

of DNA damage. **(iii)** The cut in the *AGA* gene does not produce DNA:RNA hybrids despite its transcriptional activity (by 4sU RPM) lying between *ASXL1* and *CCBL2*. **(iv)** The AsiSI cut in the *TRIM37* gene is located at a transcriptionally active site around the TSSs of three genes, with activity comparable to the previous loci. DNA:RNA hybrids do not form at this cut site following DNA damage.

Supplementary Figure 16

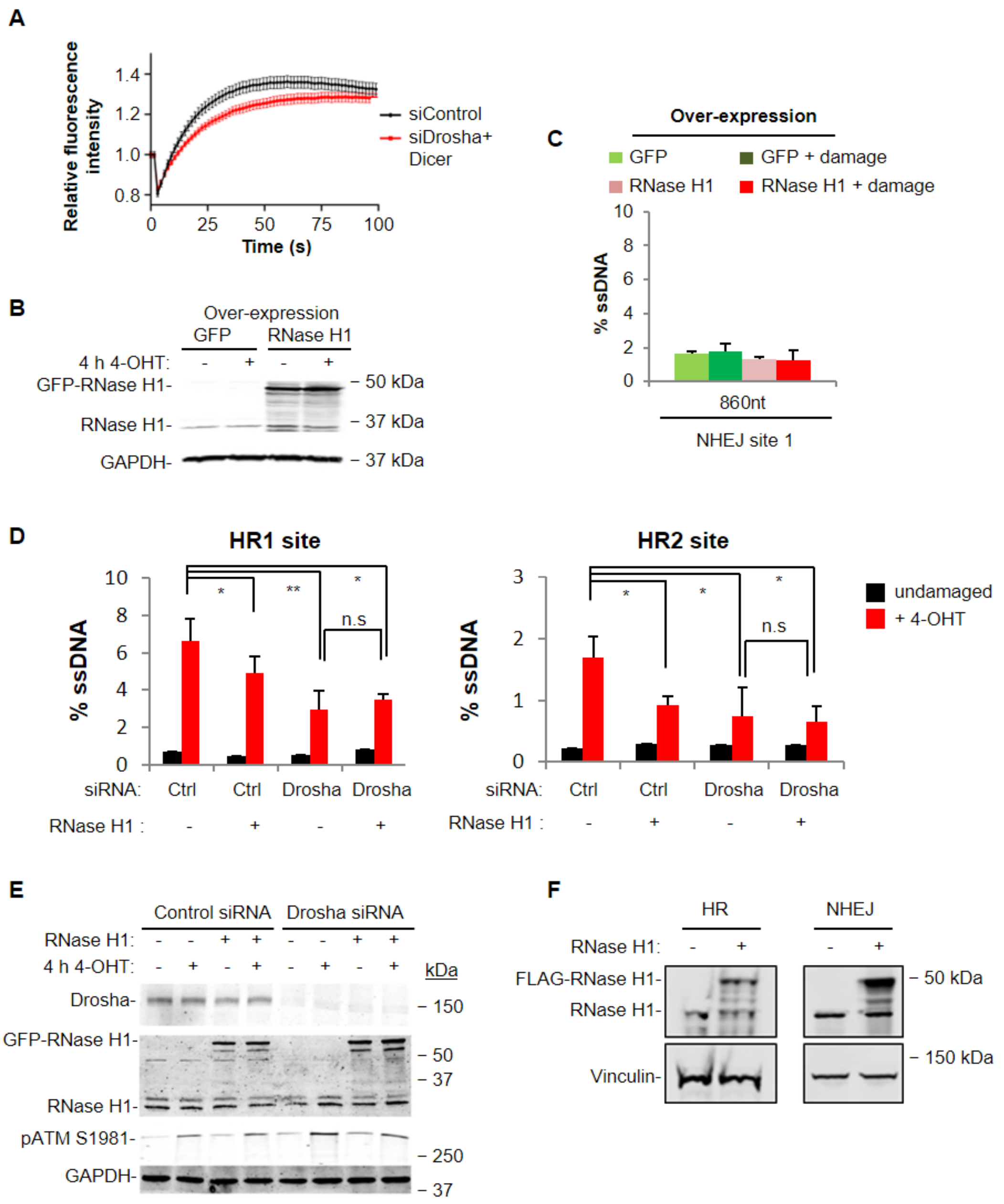

Supplementary Figure 16.

RNase H1 over-expression affects DNA repair.

(A) Relocation of inactivated *E.coli* mCherry-RNase H1 D10R E48R to sites of laser-induced DNA damage in cells treated with control or Drosha and Dicer siRNA. Graph showing quantitation of 106 cells for the control and 104 cells for the Drosha and Dicer depleted conditions over 3 replicates, error bars=SEM.

(B) Verification of RNase H1 over-expression corresponding to Fig.7D. U2OS-HA-ER-AsiSI cells were transfected with either GFP or a GFP-tagged mammalian RNase H1. After 48 hours, DNA damage was induced for 4 hours by the addition 300 nM 4-OHT. Expression of the RNase H1 construct is shown by blotting with an anti-RNase H1 antibody.

(C) GFP-RNase H1 over-expression does not significantly change the resected DNA at an NHEJ site after DNA damage. Histogram supports Fig.7D, showing DNA resection at NHEJ-prone sites is not affected by RNase H1 over-expression. The experiment is performed as in Fig.7D on the same samples. Results are of 3 biological repeats, error bars=SD.

(D) DNA resection assay as in Fig. 5 and S10AC, Drosha depletion reduces resection at two HR prone loci (HR1 131nt, HR2 615 nt from break, respectively), as does RNase H1 over-expression. The combination of both does not have an additive effect, suggesting that Drosha and R-loops function within the same pathway and that Drosha performs a role before resection occurs. Results are of 3 biological repeats, error bars =SD, paired Student's t-test, *$p<0.05$, **$p<0.01$.

(E) Western blot validation for part (D).

(F) Confirmation of over-expression of RNase H1 in the HR (left) and NHEJ (right) reporter cell lines in figure 7E.

Supplementary Figure 17

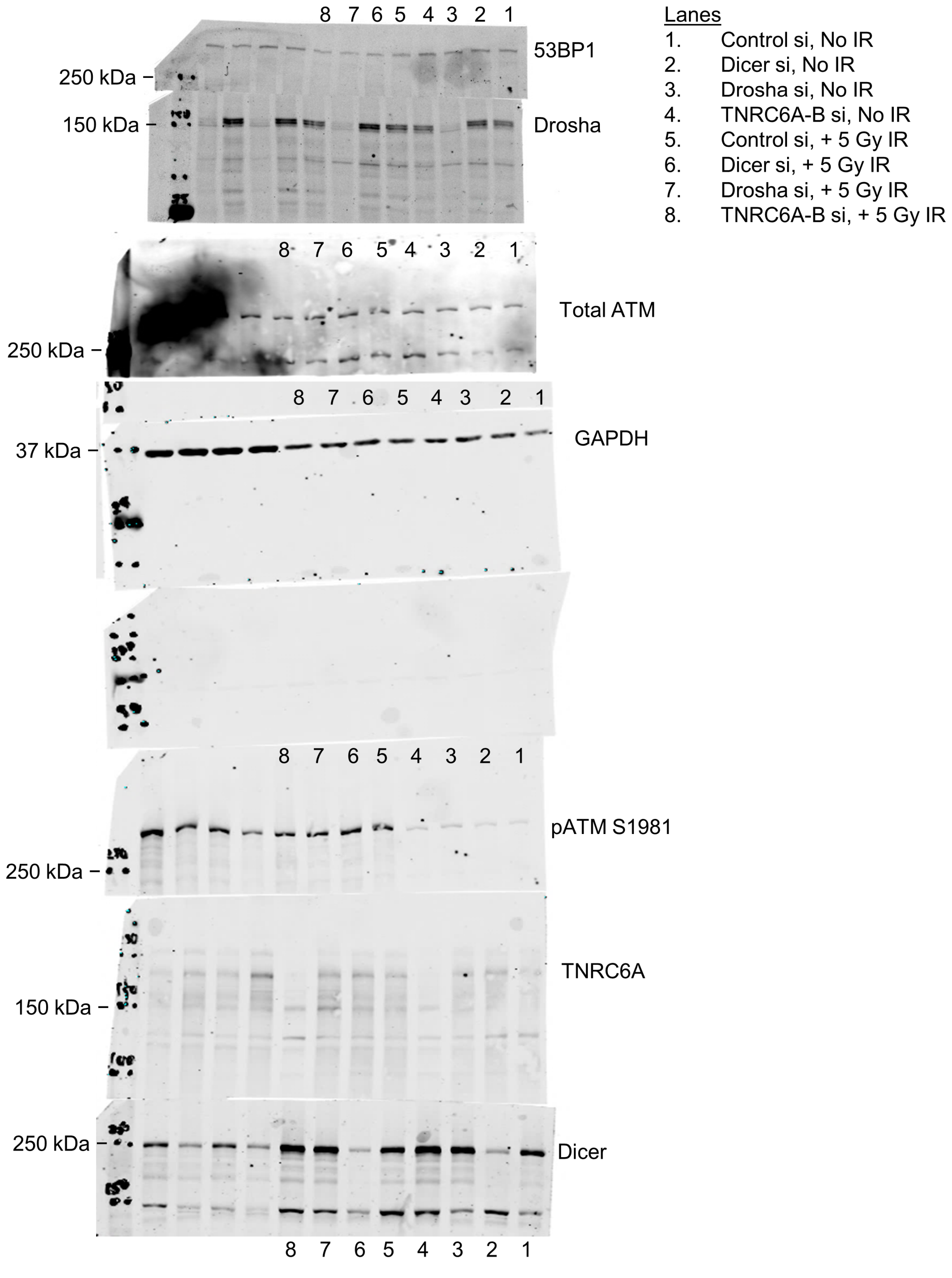

Supplementary Figure 17.

Uncropped main figure blots. Uncropped LI-COR images as scanned for figure 1C. Lanes are indicated by the numerical key. Blots were cropped and re-orientated for the final figure.

Supplementary Table 1. List of antibodies used

Antibody	Applications	Supplier	Catalogue no.	Dilution
Drosha	ChIP	Abcam	ab12286	4ug per sample
Histone H3	ChIP	Abcam	ab1791	4ug per sample
IgG	ChIP	Novus Biological	nb810-56910	4ug per sample
53BP1	IF	Abcam	ab36823	1:600
BRCA1	IF	Santa Cruz	sc6954	1:50
γH2A.X	IF	Cell Signaling	9718s	1:500
γH2A.X	IF	Millipore	05-636	1:600
MDC1	IF	Abcam	ab11169	1:600
phospho ATM Ser1981	IF	Cell Signaling	#4526	1:500
Rad51	IF	Santa Cruz	sc8349	1:50
RNF168	IF	Millipore	ABE367	1:100
Conjugated ubiquitin (clone FK2)	IF	Cayman Chemical	14220	1:200
53BP1	WB	Abcam	ab36823	1:2000
Ago2	WB	Abcam	ab32381	1:2000
ATM	WB	Santa Cruz	sc23921	1:500
Chk1	WB	Cell Signaling	#2360	1:1000
Chk2	WB	Cell Signaling	#2662P	1:1000
Dicer	WB	Santa Cruz	sc30226	1:500
Drosha	WB	Santa Cruz	sc39591	1:500
GAPDH	WB	Santa Cruz	sc32233	1:3000
GFP	WB	Abcam	ab13970	1:1000
HA	WB	Roche	1867423001	1:500
phospho ATM Ser1981	WB	Abcam	ab81292	1:2000
phospho Chk1 S3er45	WB	Cell Signaling	2348p	1:1000
phospho Chk2 Thr68	WB	Cell Signaling	#2661	1:1000
RNase H1	WB	Proteintech	1506-1-ap	1:1000
TNRC6A	WB	Novus Biological	NBP1-28757	1:1500
Vinculin	WB	Abcam	ab18058	1:3000

Supplementary Table 2. List of primers used for ssDNA resection assay.

Loci (hg38)	Restriction enzyme used	Distance from DSB (nt)	Primer sequence
HR site 1<br>Chr22:38468100	BanI	-247	FWD: CTTCCCTCCGCAGAAACTG<br>REV: CCGGGAAGATGAGGACAATA
		131	FWD:ACCATGAACGTGTTCCGAAT<br>REV:GAGCTCCGCAAAGTTTCAAG
		851	FWD:ACAGATCCAGAGCCACGAAA<br>REV:CCCACTCTCAGCCTTCTCAG
		1532	FWD:CCCTGGTGAGGGGAGAATC<br>REV: GCTGTCCGGGCTGTATTCTA
HR site 2<br>Chr20:32358513	BanI	615	FWD:GTCCCCTCCCCCACTATTT<br>REV:ACGCACCTGGTTTAGATTGG
		2001	FWD:GTTCCTGTTATGCGGGTGTT<br>REV:TGGACCCCAAATTCCTAAAG
HR site 4<br>chr1: 88992917	BsrGI-HF	245	FWD:GAATCGGATGTATGCGACTGATC<br>REV: TTCCAAAGTTATTCCAACCCGAT
		1522	FWD: TGAGGAGGTGACATTAGAACTCAGA<br>REV: AGGACTCACTTACACGGCCTTT
		3424	FWD: TCCTAGCCAGATAATAATAGCTATACAAACA<br>REV: TGAATAGACAGACAACAGATAAATGAGACA
NHEJ site1<br>chr6:89638470	BamHI-HF	860	FWD:GGCACCTCAACAGGTAGCAT<br>REV:GCCTCTCTTCGATGCTTTTG
		2600	FWD:GGAAATGAGTCGGAGACAGC<br>REV:TGCCCTCATATTCAGTGTGC
NHEJ site2<br>chr9:29212805	PstI-HF	617	FWD:GATGAGGCGGAAAGGTGTAA<br>REV:CCTCCTCAAAGCCTCCTCAC
		1087	FWD:CGAGAGGCCACCAGACTAAA<br>REV: TTCACGAAGCGATGTGTAGG
No DSB	HindIII-HF	N/A	FWD:ATTGGGTATCTGCGTCTAGTGAGG<br>REV: GACTCAATTACATCCCTGCAGCT

Supplementary Table 3. List of primers used for DRIP, ligation-mediated DNA cleavage assay and ChIP.

Loci	Primer sequence	Amplicon (hg38)
HR1	FWD:CCGCCAGAAAGTTTCCTAGA<br>REV:CTCACCCTTGCAGCACTTG	chr22:38468175-38468329
HR2	FWD:CCTAGCTGAGGTCGGTGCTA<br>REV:GAAGAGTGAGGAGGGGGAGT	chr20:32358116-32358311
HR3	FWD:GAGGAGCGCAGGACACTG<br>REV:CCAATTAGAGACCACCCGTTT	chr17:82292628-82292820
HR4	FWD: CTCTAGGCTGGAAGAGACTG<br>REV: AGCCCGGAACTGCCCAAATC	chr1:88992742-88992815
HR5	FWD: GTCCCTCGAAGGGAGCAC<br>REV: CCGACTTTGCTGTGTGACC	chr9:127930978-127931177
NHEJ1	FWD:ATCGGGCCAATCTCAGAGG<br>REV:GCGACGCTAACGTTAAAGCA	chr6:89638541-89638692
NHEJ2	FWD:GGTGCCCACAGCTCTCTATG<br>REV:GAAGCCAGAGGAGTGTCCTG	chr9:29212545-29212742
γActin exon5	FWD:GTGACACAGCATCACTAAGG<br>REV:ACAGCACCGTGTTGGCGT	chr17:81511013-81511146
miR-122 exon2	FWD:ACATGCCTGTGTCCACTGCT<br>REV:CTCTAGGTGGCCCCAGTCAC	chr18:58449658-58449729
Plasmid spike in control for cleavage assay	FWD: TCGAGAGCAACTGGAATGAG<br>REV: AAGCATAGATGCCACGAAGG	
